# Caraway Extract Increases *Ucp-1* mRNA Expression in C3H10T1/2 Adipocytes Through Direct and Indirect Effects

**DOI:** 10.3390/ijms262210970

**Published:** 2025-11-12

**Authors:** Hisako Takahashi, Nanami Tomishima, Toshihiro Suzuki, Hiromu Morimoto, Hirofumi Inoue, Kentaro Kaneko, Tsuyoshi Goto, Teruo Kawada, Mariko Uehara, Nobuyuki Takahashi

**Affiliations:** 1 Department of Agricultural Chemistry, School of Agriculture, Meiji University, 1-1-1 Higashimita, Tama-ku, Kawasaki-shi 214-8571, Kanagawa, Japan; hisako4132@icloud.com (H.T.);; 2 Department of Nutritional Science and Food Safety, Faculty of Applied Bioscience, Tokyo University of Agriculture, 1-1-1 Sakuragaoka, Setagaya-ku, Tokyo 156-8502, Japan; 3 Department of Fermentation Science, Faculty of Applied Bioscience, Tokyo University of Agriculture, 1-1-1 Sakuragaoka, Setagaya-ku, Tokyo 156-8502, Japan; 4 Division of Food Science and Biotechnology, Graduate School of Agriculture, Kyoto University, Kitashirakawa Oiwake-cho, Sakyo-ku, Kyoto 606-8502, Japan; goto.tsuyoshi.6x@kyoto-u.ac.jp (T.G.);

**Keywords:** brown adipocytes, beige adipocytes, UCP-1, caraway, adrenergic sensitivity

## Abstract

*Carum carvi*, commonly known as caraway, is a medicinal and culinary plant recognized for its anti-inflammatory properties, primarily attributed to its essential oil components. However, the thermogenic potential of caraway—particularly the biological activity of its water-soluble extract—remains largely unexplored. In this study, we investigated the effects and underlying mechanisms of caraway on *Ucp-1* mRNA expression in beige adipocytes and on inflammation-mediated suppression of thermogenesis, by treating C3H10T1/2 adipocytes with caraway water extract (CWE) or caraway hexane extract (CHE) during both the induction and maturation phases, followed by isoproterenol stimulation, and measurement of mRNA levels of *Ucp-1* and differentiation-related genes. Additionally, RAW264.7 cells were treated with CWE prior to stimulation with lipopolysaccharides followed by evaluation of inflammatory marker expression. CWE increased *Ucp-1* mRNA expression directly by enhancing adrenergic sensitivity and promoting beige adipocyte differentiation during the induction phase of differentiation. Further, CWE mediated an indirect effect on *Ucp-1* expression by suppressing macrophage inflammation, thus restoring *Ucp-1* expression otherwise inhibited under inflammatory conditions. These results suggest that caraway extracts—especially the water-soluble compounds—may serve as therapeutic candidates for obesity-related conditions by enhancing energy expenditure and mitigating chronic inflammation.

## 1. Introduction

Brown and beige adipocytes are thermogenic cells that play a crucial role in energy expenditure by dissipating the proton gradient across the inner mitochondrial membrane via uncoupling protein-1 (UCP-1), thereby generating heat instead of ATP [1]. Increased UCP-1 expression in mouse adipose tissue suppresses fat accumulation and improves metabolism [2]. Furthermore, in humans, activation of brown adipose tissue contributes to increased energy expenditure [3]. Consequently, promoting UCP-1 expression in adipocytes has emerged as a promising strategy for treating obesity and related metabolic disorders [4,5]. However, the thermogenic capacity of adipose tissue can be attenuated under chronic inflammatory conditions that are prevalent in obesity. Proinflammatory cytokines such as tumor necrosis factor-alpha (TNFα) and interleukin-1 beta (IL-1β) regulate UCP-1 expression, thereby affecting the process of energy dissipation [6,7,8]. Previous studies investigating food-derived compounds that promote UCP-1 expression have often focused solely on effects at the basal expression level, without considering conditions such as adrenergic stimulation [9,10,11]. Consequently, the potential of these compounds to enhance UCP-1 expression under physiologically relevant stimuli that mimic in vivo thermogenic activation remains to be elucidated. Furthermore, these food-derived compounds are lipid-soluble, among which spices, in particular, have been studied for their essential oil components [10,12,13,14,15]. In contrast, water-soluble compounds derived from medicinal and edible plants have received comparatively limited attention, despite their potential to exhibit unique biological effects via various mechanisms.

*Carum carvi* (*C. carvi*), commonly known as caraway, is a medicinal and culinary plant recognized for its anti-inflammatory properties, which are primarily attributed to its essential oils [16,17,18]. Caraway has been shown to reduce colitis in animals [19]. However, the efficacy of caraway has been reported for essential oils and lipid-soluble extracts, with limited data regarding its water-soluble extracts. Furthermore, its potential thermogenic effects have not been investigated. Evaluating the activity of water-soluble extracts is crucial as they are commonly extracted using water or hot water during culinary preparation, in addition to oil-based methods. Therefore, this study examined the effects of both hexane and water extracts of caraway on *Ucp-1* mRNA expression in C3H10T1/2 adipocytes and on inflammatory responses in RAW264.7 macrophages. Our findings indicate that caraway water extract (CWE) increases *Ucp-1* mRNA expression through two distinct mechanisms: (1) a direct effect that enhances adrenergic sensitivity and promotes beige adipocyte differentiation when applied during the induction phase of differentiation, and (2) an indirect effect mediated by suppression of macrophage inflammation, which restores *Ucp-1* mRNA expression suppressed under inflammatory conditions. These results suggest that caraway extracts, particularly their water-soluble components, could serve as functional food ingredients to prevent obesity-associated metabolic dysfunction by promoting thermogenesis and alleviating chronic inflammation.

## 2. Results

### 2.1. C. carvi Extract Enhances UCP-1 Expression by Increasing Adrenergic Sensitivity in C3H10T1/2 Adipocytes

**Figure 1:**
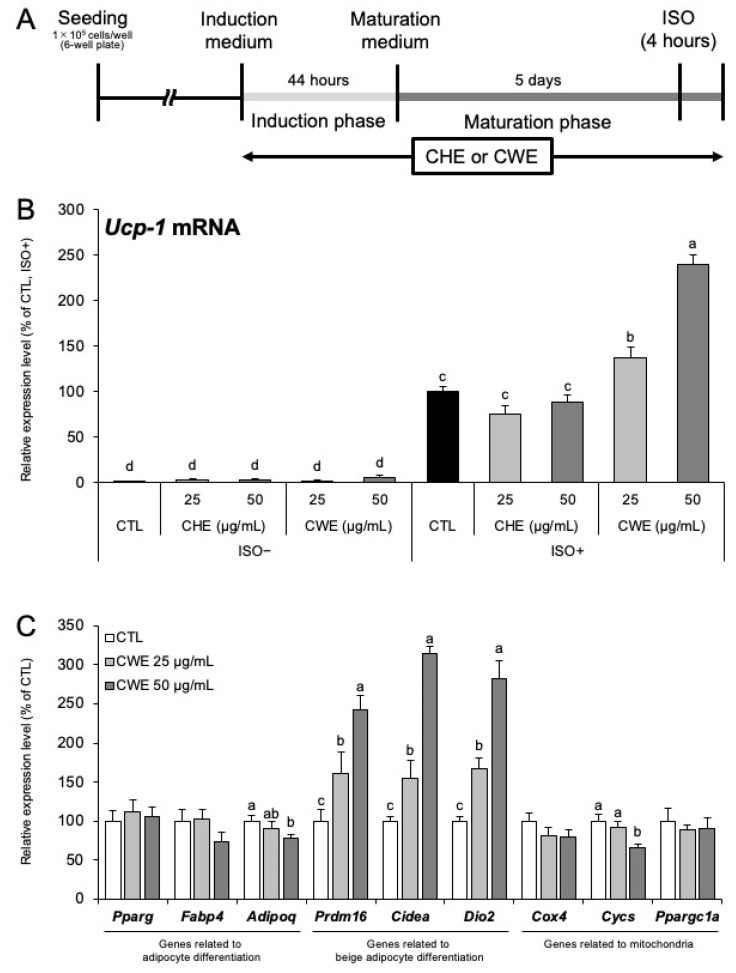
Analysis of mRNA expression in C3H10T1/2 adipocytes treated with *C. carvi* extracts (CHE or CWE). (**A**) Diagram showing the experimental timeline. After treatment with either hexane extract (CHE) or water extract (CWE) during the induction and maturation phases, cells received isoproterenol (ISO) treatment for 4 h. (**B**) *Ucp-1* mRNA expression with ISO stimulation: mRNA expression of *Ucp-1* in C3H10T1/2 cells treated with CHE or CWE. The control group, which received ISO but not the extract, was set as 100%, and the relative values of other groups are shown. (**C**) mRNA expression in CWE-treated cells (without ISO stimulation). mRNA expression in C3H10T1/2 cells treated with CWE. Only the group without ISO stimulation was included. The extract-untreated group was used as the control, set at 100%, and the relative values of the extract-treated groups are shown. Each bar represents the mean ± S.D. (*n* = 3). Different letters indicate statistically significant differences between groups (*p* < 0.05).

We evaluated the effects of the hexane (CHE) and water (CWE) extracts of *C. carvi* on *Ucp-1* mRNA expression in C3H10T1/2 cells. First, we assessed the cytocompatibility of both extracts and found that concentrations of up to 50 µg/mL were non-toxic for both CHE and CWE (Figure S1A,B). C3H10T1/2 cells were treated with CHE or CWE for 7 days (Figure 1A) and stimulated with the adrenergic agonist isoproterenol (ISO) for 4 h to induce *Ucp-1* expression. Neither CHE nor CWE affected *Ucp-1* mRNA expression in the absence of ISO stimulation. In contrast, under ISO stimulation, CHE had no effect on *Ucp-1* mRNA levels, whereas CWE significantly increased *Ucp-1* mRNA in a dose-dependent manner (Figure 1B). *Ppargc1a* mRNA also increased under ISO stimulation (Figure S2). Furthermore, the effect of caraway on increasing *Ucp-1* mRNA expression was maintained under hot water extraction (CHWE), acetic acid extraction (CAE), heating treatment (HT-CWE), and acetic acid treatment (AA-CAE) (Figure S3). CWE treatment did not alter *Ucp-1* mRNA expression before ISO stimulation, indicating that the *Ucp-1* expression-enhancing effect of CWE manifests exclusively under ISO-induced adrenergic stimulation.

The expression levels of adipocyte differentiation marker genes (*Pparg*, *Fabp4*, and *Adipoq*), beige adipocyte marker genes (*Prdm16*, *Cidea*, and *Dio2*), and genes related to mitochondrial biogenesis and function (*Cox4*, *Cycs*, and *Ppargc1a*) were analyzed using RT-qPCR in C3H10T1/2 cells treated with CWE without ISO stimulation. The mRNA expression levels of *Prdm16*, *Cidea*, and *Dio2* exhibited a dose-dependent increase in response to CWE (Figure 1C). This effect was also confirmed under ISO stimulation (Figure S4). The expression of adipocyte differentiation marker genes and genes associated with mitochondrial function has not increased. In addition, CWE had no effect on lipid accumulation (Figure S5A,B).

### 2.2. CWE Enhances Adrenergic Sensitivity During the Induction Phase of Adipocyte Differentiation

**Figure 2:**
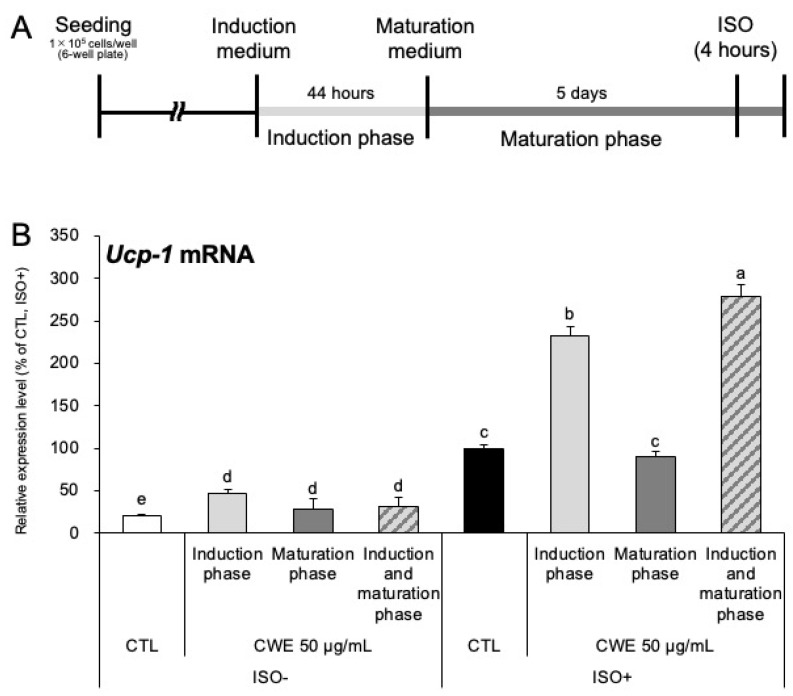
*Ucp-1* mRNA expression analysis in CWE-treated C3H10T1/2 adipocytes. (**A**) Diagram showing the experimental timeline. After treatment with CWE during the induction or maturation phase, cells were treated with ISO (ISO+) or continued in culture without ISO (ISO−) for 4 h. (**B**) *Ucp-1* mRNA expression in C3H10T1/2 cells treated with CWE. The control group, which received only ISO but no CWE, was set as 100%. Each bar represents the mean ± S.D. (*n* = 3). Different letters indicate statistically significant differences between groups (*p* < 0.05).

As shown in Section 2.1, CWE enhances adrenergic sensitivity in C3H10T1/2 adipocytes, leading to increased *Ucp-1* mRNA expression in response to ISO stimulation. Adipocyte differentiation is a multistep process that begins with a 44 h stimulation period (the induction phase), using an induction medium to trigger the early differentiation steps. This is followed by the maturation phase, supported by a maturation medium, which facilitates the formation and maturation of fat droplets. To pinpoint the active stage, we treated cells with CWE separately during the induction or maturation phase (Figure 2A). CWE increased adrenergic sensitivity only when administered during the induction phase (Figure 2B). At this time, no change was observed in the expression of β3 adrenergic receptor (*Adrb3*) mRNA (Figure S6A). Furthermore, analysis of the cAMP and CREB pathway—a representative pathway linking adrenergic stimulation to *Ucp-1* expression—revealed no effect on cAMP levels, CREB phosphorylation, or transcriptional activity (Figure S6B–D).

### 2.3. CWE Promotes Beige Adipocyte Differentiation During the Induction Phase

**Figure 3:**
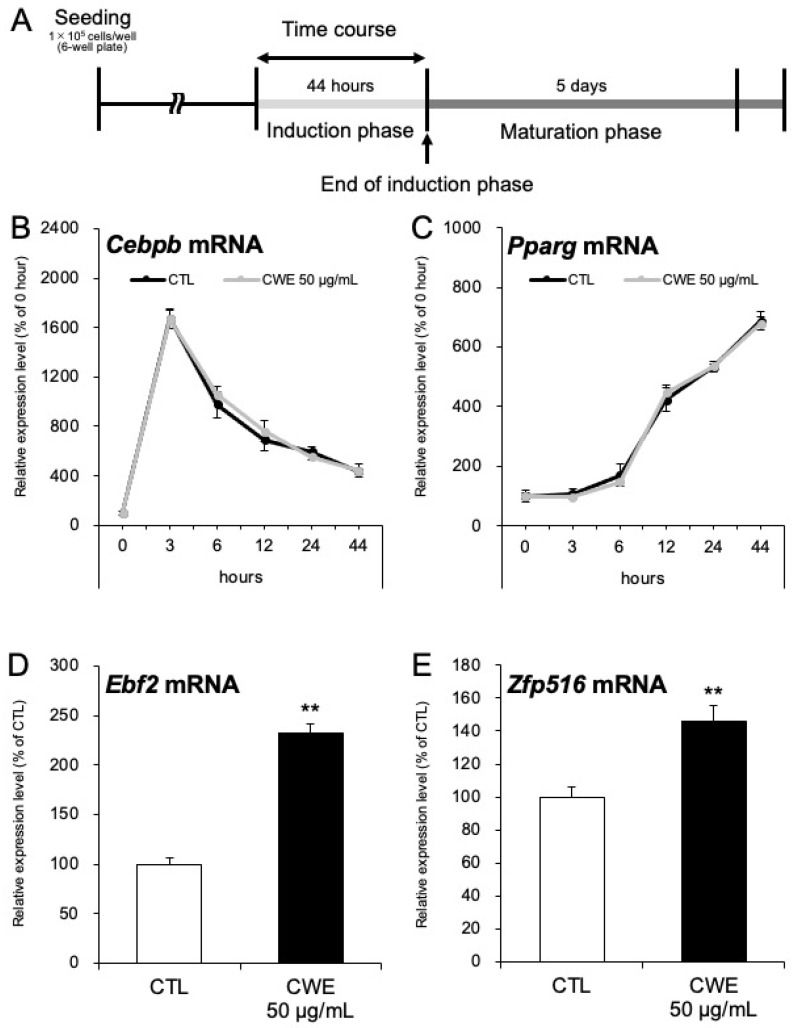
mRNA expression analysis in C3H10T1/2 adipocytes treated with CWE in the induction phase. (**A**) Diagram showing the experimental timeline. CWE was administered only during the induction phase. (**B**,**C**) mRNA expression of *Cebpb* (**B**) and *Pparg* (**C**) in C3H10T1/2 cells treated with CWE. mRNA expression was analyzed at multiple time points in the induction phase: 3, 6, 12, 24, and 44 h (corresponding to the “Time course” section in Figure 3A). The 0 h control was set as 100%, and relative expression levels were calculated accordingly. (**D**,**E**) mRNA expression of *Ebf2* (**D**) and *Zfp516* (**E**) in C3H10T1/2 cells treated with CWE in the induction phase (corresponding to the “End of induction phase” section in Figure 3A). The extract-untreated group was used as the control, set at 100%, and the relative values of the extract-treated groups are shown. Each bar represents the mean ± S.D. (*n* = 3); The significance is indicated by ** (*p* < 0.01).

As demonstrated in Section 2.2, CWE enhances adrenergic sensitivity in C3H10T1/2 adipocytes during the induction phase. To further investigate CWE’s effects during this critical period, we analyzed the expression of differentiation-related genes at multiple time points throughout the induction phase (Figure 3A). The mRNA expression of *Cebpb* (an early differentiation marker) and *Pparg* (a late differentiation marker) was not significantly different between the control and CWE-treated groups across the time points analyzed (Figure 3B,C). However, at the end of the induction phase (44 h), we further examined the expression of beige adipocyte markers. CWE treatment significantly increased the expression of *Ebf2* and *Zfp516*, which are considered early and mid-stage markers of beige adipocyte differentiation, respectively (Figure 3D,E).

### 2.4. CHE and CWE Inhibit AP-1/NF-κB Activity and Suppress Inflammatory Gene Expression in RAW264.7 Macrophages

**Figure 4:**
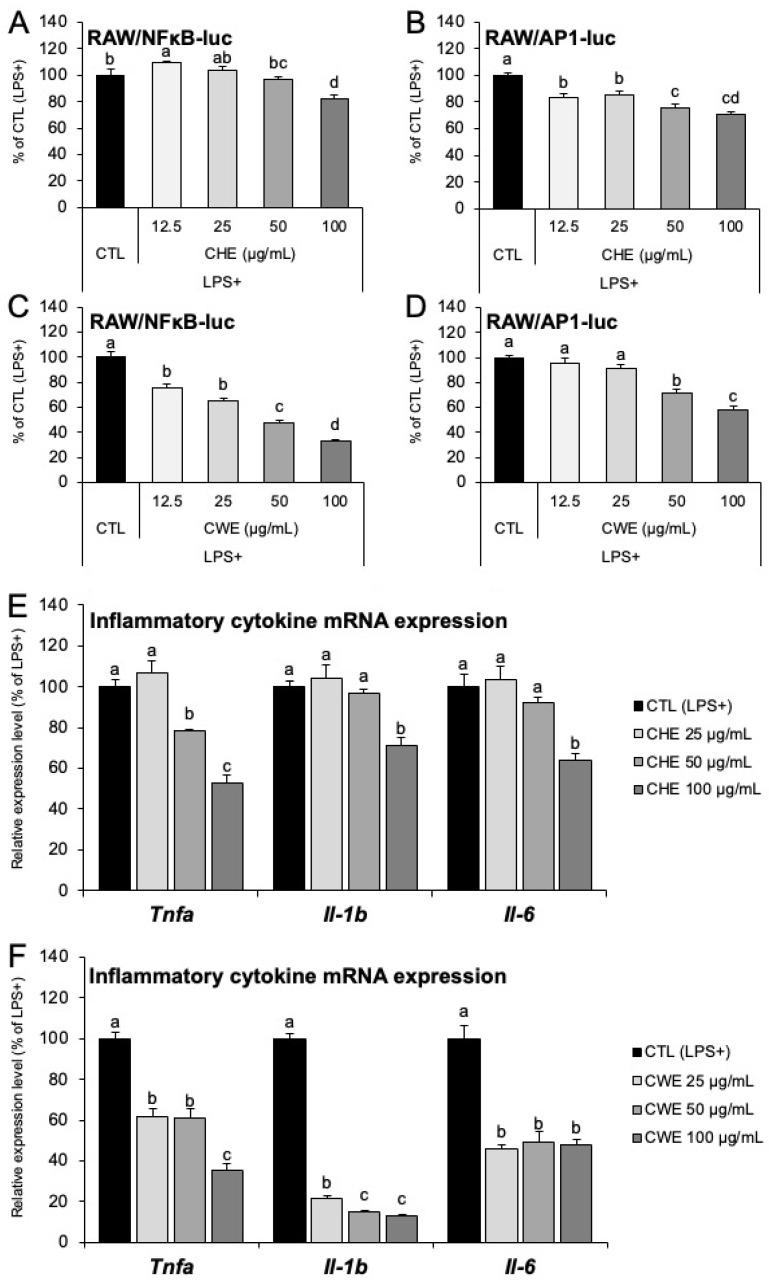
Analysis of anti-inflammatory effects of CHE or CWE on RAW 264.7 macrophages. (**A**–**D**) NF-κB and AP-1 activities were assessed using RAW/NFκB-luc and RAW/AP1-luc reporter cells. Anti-inflammatory effects of CHE are shown in panels (**A**,**B**). Anti-inflammatory effects of CWE are shown in panels (**C**,**D**). The LPS- and extract-untreated groups serve as the control, and relative values are expressed as 100% relative to this control. (**E**,**F**) mRNA expression of inflammatory cytokines in RAW264.7 cells treated with CHE and CWE. The LPS-stimulated group without CHE/CWE treatment was used as the control, and relative values are expressed as 100%. Each bar represents the mean ± S.D. (*n* = 3). Different letters indicate statistically significant differences between groups (*p* < 0.05).

To assess the effect of CHE and CWE on inflammatory gene expression in RAW264.7 macrophages, we first confirmed their cytocompatibility. Concentrations up to 100 µg/mL were non-toxic for both CHE and CWE (Figure S7A,B). We initially evaluated the anti-inflammatory effects using RAW/NFκB-luc and RAW/AP1-luc cells, which are RAW264.7 cells engineered to express respective reporter plasmids. Treatment with 100 µg/mL of CHE led to a reduction in NF-κB activity (Figure 4A). Further, AP-1 activity exhibited a dose-dependent reduction at CHE concentrations of ≥12.5 µg/mL (Figure 4B). CWE demonstrated a dose-dependent reduction in NF-κB activity, with concentrations of ≥12.5 µg/mL showing significant suppression (Figure 4C). AP-1 activity was reduced in a dose-dependent manner at concentrations of ≥50 µg/mL (Figure 4D). Following the reporter assays, we determined the effects of CHE and CWE on the mRNA expression of inflammatory cytokines. Both CHE and CWE suppressed mRNA expression of *Tnfa*, *Il-1b*, and *Il-6* at concentrations ranging from 25 to 100 µg/mL (Figure 4E,F). When evaluating the anti-inflammatory effects of other extracts—CHWE, CAE, HT-CWE, and AA-CWE—using the same method, only CAE showed no anti-inflammatory effects (Figure S8A,B). We further studied the effect of CHE, carvone, and limonene on the expression of inflammatory cytokines in lipopolysaccharide (LPS)-induced RAW264.7 macrophages. Neither carvone nor limonene had any inhibitory effects individually or in combination, whereas CHE showed significant inhibitory effects (Figure S10A). However, using RAW/NFκB-luc cells, luciferase reporter assays indicated that when macrophages were treated with LPS, carvone had significant inhibitory effects on expression at certain concentrations, whereas limonene had no such effects (Figure S10B,C). The GC-MS results of CHE showed more peaks than previously reported, suggesting that compounds other than carvone and limonene may contribute to the anti-inflammatory effects (Figure S9A).

### 2.5. Anti-Inflammatory Effects of C. carvi Extracts Restore UCP-1 Expression Suppressed by Inflammation

**Figure 5:**
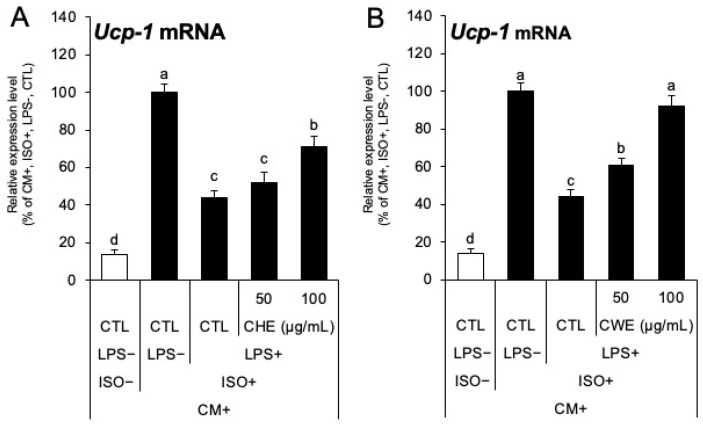
*Ucp-1* mRNA expression analysis when C3H10T1/2 cells were treated with conditioned medium (CM) from RAW264.7 cells. (**A**) CM prepared from CHE-treated RAW264.7 cells was added to C3H10T1/2 adipocytes. The black bars indicate the groups that received ISO stimulation. The control group, used to set the 100% mRNA expression level, received ISO stimulation after adding CM prepared from RAW264.7 cells not stimulated with LPS. (**B**) CM prepared from CWE-treated RAW264.7 cells was added to C3H10T1/2 adipocytes. The black bars indicate the groups stimulated with ISO. The control group, used to set the 100% mRNA expression level, received ISO stimulation after adding CM prepared from RAW264.7 cells not stimulated with LPS. Each bar represents the mean ± S.D. (n = 3). Different letters indicate statistically significant differences between groups (*p* < 0.05).

Section 2.4 demonstrated that both CHE and CWE have anti-inflammatory effects. We therefore investigated whether these anti-inflammatory properties could restore *Ucp-1* expression, which is typically suppressed by LPS-induced inflammation, by culturing C3H10T1/2 adipocytes with conditioned medium (CM) from RAW264.7 cells (the CM preparation procedure is detailed in Section 4.1). *Ucp-1* expression was suppressed in C3H10T1/2 adipocytes after LPS treatment (CTL LPS− ISO+ versus CTL LPS+ ISO+), which was partially restored in C3H10T1/2 adipocytes cultured in CM prepared from RAW264.7 cells treated with 100 µg/mL CHE (Figure 5A). The expression was almost completely restored in a dose-dependent manner in C3H10T1/2 adipocytes treated with CM prepared from RAW264.7 cells treated with 50 and 100 µg/mL CWE (Figure 5B).

## 3. Discussion

In this study, we showed that CWE increases *Ucp-1* mRNA expression in C3H10T1/2 adipocytes through both direct and indirect effects. This activity was not observed with CHE, suggesting that the primary active compound(s) are water-soluble. Generally, increased *Ucp-1* expression in adipocytes implies enhanced thermogenic capacity, which is considered an anti-obesity effect [20]. As shown in Figure 1B, the increase in *Ucp-1* expression occurred only under adrenergic stimulation with ISO, suggesting that CWE enhances adrenergic sensitivity rather than inducing basal *Ucp-1* expression. Notably, CWE selectively increased the expression of beige adipocyte marker genes (*Prdm16*, *Cidea*, and *Dio2*) but did not affect general adipogenic differentiation markers (*Pparg*, *Fabp4*, and *Adipoq*) or mitochondrial-related genes (*Cox4*, *Cycs*, and *Ppargc1a*) (Figure 1C,D). This selective effect is supported by the unaltered lipid accumulation observed in the cells (Figure S5A,B). The activation of *Ucp-1* transcription is significantly influenced by PR domain containing 16 (PRDM16) [21,22]. The increased expression of *Prdm16* likely contributes to the elevated *Ucp-1* levels. Similarly, Cell death–inducing DNA fragmentation factor, alpha subunit-like effector A (CIDEA) and deiodinase, iodothyronine, type II (DIO2) are specifically increased during beige adipocyte differentiation [23,24,25]. Their upregulation indicates that the adipocytes are in a state more conducive to *Ucp-1* expression. This evidence strongly suggests that CWE selectively induces beige adipocyte differentiation rather than promoting general adipogenesis. Furthermore, the results shown in Figure 2A,B indicate that CWE’s action is specific to the induction phase of adipocyte differentiation, implying its involvement in early transcriptional regulatory events that direct precursor cells toward a beige phenotype.

To investigate the mechanisms by which CWE enhances adrenergic sensitivity, we analyzed the representative cascade leading from adrenergic stimulation to *Ucp-1* expression. This pathway begins with norepinephrine binding to the β3 adrenergic receptor (Adrb3), which increases intracellular cAMP concentration, activating PKA, and subsequently phosphorylating and activating CREB to initiate transcription [26]. We first analyzed *Adrb3* mRNA levels but observed no change following CWE treatment (Figure S6A). Next, we examined intracellular cAMP concentration after ISO stimulation, which was not altered by CWE (Figure S6B). Finally, no changes in CREB activity were observed with CWE treatment across any experimental method (Figure S6C,D). These findings suggest that CWE does not activate the major Adrb3-to-CREB pathway for *Ucp-1* expression. Isolating the active compounds from the extract would enable a more detailed investigation of the underlying mechanisms.

Early B cell factor 2 (EBF2) is an established early differentiation marker of beige adipocytes [27,28], while ZFP516 is a mid-stage marker [29]. The increased expression of both *Ebf2* and *Zfp516* suggests that CWE acts upstream of PRDM16, potentially enhancing the commitment to beige adipocyte differentiation. EBF2 influences the chromatin structure at the binding site for PGC1-α (a transcription regulator of *Ucp-1* activated by adrenaline), thereby facilitating PGC1-α binding [26,27,28]. Thus, the increased *Ebf2* expression further supports the claim that CWE enhances adrenaline sensitivity. While we have previously reported that food-derived compounds can enhance adrenaline sensitivity [30], the mechanism remained unclear. The present study provides direct evidence that increased *Ebf2* expression may upregulate *Ucp-1* expression via enhanced adrenaline.

Based on the observation that CWE does not alter *Adrb3* expression or CREB activity (Figure S6A,C,D) but increases *Ebf2*, *Zfp516* (Figure 3D,E), and *Prdm16* (Figure 1C) expression, we anticipate that increased *Ebf2* contributes to stabilizing chromatin structure, while increased *Zfp516* and *Prdm16* lead to the formation of more transcription complexes, ultimately increasing *Ucp-1* mRNA expression during adrenergic stimulation (Figure S11).

CWE also exhibited strong anti-inflammatory effects in RAW264.7 macrophages. Both CHE and CWE reduced the transcriptional activity of NF-κB and AP-1 (Figure 4A–D) and significantly suppressed the mRNA expression of inflammatory cytokines (Figure 4E,F), suggesting that both extracts may exert anti-inflammatory effects by acting upstream of NF-κB and AP-1. Notably, carvone and limonene, major compounds of CHE, did not show any anti-inflammatory effects [16,18] at the concentrations present in the CHE used in this study (Figure S10A–C). Thus, the anti-inflammatory effect of CHE is likely due to compounds other than carvone and limonene. Since CHE and CWE contain different compounds [31,32], the active anti-inflammatory compounds in CWE are also likely distinct. Due to the large number of compounds in CWE, individual compound analysis was beyond the scope of this study; further investigation of the hydrophilic compounds is necessary.

**Figure 6:**
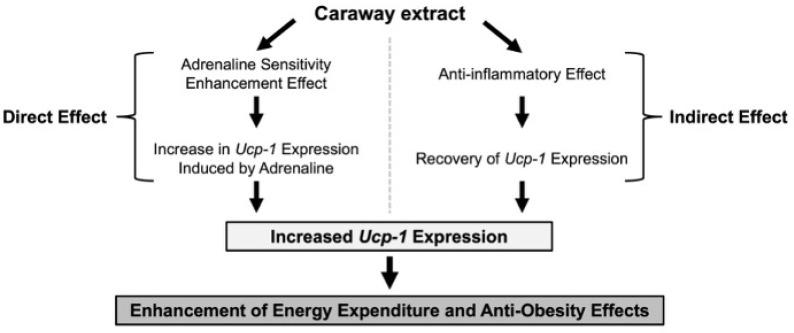
Direct and indirect enhancement of *Ucp-1* expression by caraway extract leads to anti-obesity effects.

As the physiological significance of the anti-inflammatory effect was established, we further examined its impact on thermogenesis. We found that the CM obtained from macrophages treated with CHE or CWE restored *Ucp-1* expression in adipocytes that had been suppressed by inflammation (Figure 5A,B). Chronic inflammation associated with obesity and metabolic disorders is known to impair heat production [7,33]. This suggests that the anti-inflammatory effects of CHE or CWE may indirectly contribute to maintaining beige adipocyte function. The combined results indicate that CWE possesses a dual mechanism for enhancing *Ucp-1* expression: (1) direct promotion of beige adipocyte differentiation during the adipocyte induction phase, and (2) indirect support through macrophage-mediated suppression of inflammation (Figure 6). Previous analyses of the physiological effects of *C. carvi* have primarily focused on lipophilic compounds such as essential oils [34]. However, this study is novel in finding that CWE increases *Ucp-1* expression via adrenaline sensitivity enhancement and restores inflammation-suppressed *Ucp-1* expression via anti-inflammatory effects. These findings expand the potential of spices and highlight the importance of investigating water-soluble extracts, particularly since the mechanism of adrenaline sensitivity enhancement has been partly elucidated through the role of *Ebf2*. The findings of this study suggest that CWE may serve as a functional ingredient with potential anti-obesity properties.

## 4. Materials and Methods

### 4.1. Chemicals

Carvone and limonene were purchased from Tokyo Chemical Industry Co., Ltd. (Tokyo, Japan). All other reagents were purchased from FUJIFILM Wako Pure Chemical Corporation (Osaka, Japan) or Nacalai Tesque, Inc. (Kyoto, Japan).

### 4.2. Cell Culture

We used the cell lines C3H10T1/2 (adipocyte model, Sumitomo Dainippon Pharmaceutical Co., Ltd., Osaka, Japan) and RAW264.7 (macrophage model, RIKEN BioResource Center, Tsukuba, Japan). C3H10T1/2 cells were maintained in high-glucose DMEM (glucose 4.5 mg/mL), whereas RAW264.7 cells were cultured in αMEM supplemented with 10% FBS, 100 U/mL penicillin, and 100 μg/mL streptomycin. Both cell lines were cultured at 37 °C in a humidified 5% CO_2_ atmosphere. C3H10T1/2 differentiation was initiated by culturing cells in an induction medium (containing 0.5 mM 3-isobutyl-1-methylxanthine, 0.25 μM dexamethasone, and 10 μg/mL insulin) for 44 h. The medium was then switched to a maturation medium (containing 5 μg/mL insulin), which was refreshed every 2 days thereafter. *Ucp-1* mRNA expression was induced by treating mature adipocytes with 10 µM ISO for 4 h at the end of the maturation phase.

### 4.3. Treatment of C3H10T1/2 Cells with Extracts

C3H10T1/2 adipocytes were treated with the respective extracts (CHE or CWE) at the indicated concentrations during the induction phase, maturation phase, or both. ISO stimulation was performed at the same time point as described in Section 4.2. Upon completion of all treatments, cells were collected for RNA isolation and gene expression measurement.

### 4.4. Treatment of RAW264.7 Macrophages with Extracts

Inflammation in RAW264.7 cells was induced using LPS. RAW264.7 cells expressing the respective luciferase reporter plasmids were pre-treated with the extract for 24 h. Inflammation was then induced by adding 0.1 µg/mL of LPS during the final 3 h of the 24 h period. Luciferase activity was measured as described in Section 4.8. RAW264.7 cells were treated with extracts for 24 h. Inflammation was induced by adding 1 µg/mL LPS during the final 6 h of the 24 h treatment. *Ucp-1* mRNA expression was measured using RT-qPCR.

### 4.5. Treatment of C3H10T1/2 Adipocytes with RAW264.7-Conditioned Medium

CM was prepared following a previously reported method [35]. RAW264.7 macrophages were pre-treated with the indicated concentrations of CWE/CHE for 24 h. During this 24 h period, the cells were stimulated with 1 μg/mL LPS for 6 h. Cells were then washed and cultured in serum-free medium for 24 h. This medium was collected, stored at 4 °C and used as CM. Differentiated C3H10T1/2 adipocytes were treated with CM for 24 h. At 20 h of culture, the cells were treated with 10 μM ISO for 4 h. Cells were then collected to measure *Ucp-1* mRNA levels using qRT-PCR.

### 4.6. Preparation of Extracts

*C. carvi (Caraway*) was sourced from the Netherlands and supplied by Camel Coffee Co., Ltd. (Japan, Tokyo). Extraction methods were based on previous reports [32]. One gram of *C. carvi* was extracted with 22 mL of n-hexane by heating at 60 °C for 2 h. The filtered extract was dried using an evaporator at 55 °C to remove n-hexane. The resultant extract was dissolved in DMSO and designated as CHE for use in experiments. The *C. carvi* residue remaining after hexane extraction was subjected to water extraction at 4 °C for 24 h. The filtered extract was lyophilized (freeze-dried) to remove water. The resulting powder was dissolved in water, and designated as CWE for cell experiments.

### 4.7. RNA Preparation and Quantitative RT-PCR Analysis

Total RNA was extracted from cultured cells using Sepasol (R)-RNA I Super (Nacalai Tesque, Inc.) following the manufacturer’s instructions. Reverse transcription (RT) was performed using a ReverTra ACE^®^ kit (TOYOBO Co., Ltd., Osaka, Japan) on a thermal cycler (LifeEco, Nippon Genetics Co., Ltd., Tokyo, Japan). Quantitative RT-PCR (qPCR) was conducted using SYBR Green fluorescent signals on the StepOnePlus system (Thermo Fisher Scientific Inc., Waltham, MA, USA) following established protocols [25]. Oligonucleotide primers were designed using the Primer-BLAST website. Primer sequences are provided in Supplementary Table S1. *36b4* mRNA expression was used as an internal control. All mRNA expression levels are presented as a ratio relative to the control in each experiment.

### 4.8. Luciferase Ligand Assay

pGL4.32 [luc2p/NFκB-RE/Hygro] and pGL4.44 [luc2P/AP1-RE/Hygro] plasmids (Promega K.K., Madison, WI, USA) were transfected into different RAW264.7 cells using Lipofectamine 2000 (Thermo Fisher Scientific Inc.). Stable expression strains were selected with 100 μg/mL hygromycin. Transfected cells were cultured in 96-well plates and treated with extracts and LPS as described in Section 4.4. Cells were then lysed, and the luciferase assay was performed using the luciferase reporter gene assay system from Promega K.K.

### 4.9. Statistical Analyses

Results are presented as the mean ± S.D. (*n* = 3 for all analyses). No experimental units were excluded. Statistical significance was determined using Tukey’s multiple comparison test following one-way analysis of variance (ANOVA). For results involving multiple factors, Tukey’s multiple comparison test was performed after two-way ANOVA when interactions were observed. The statistical significance level was set at *p* < 0.05.

## Data Availability

The datasets used are available on request from the authors.

## Supplementary Materials

The following supporting information can be downloaded at: https://www.mdpi.com/article/10.3390/ijms262210970/s1.
