# Supplementary material for: Caraway Extract Increases Ucp-1 mRNA Expression in C3H10T1/2 Adipocytes Through Direct and Indirect Effects
Source: Int J Mol Sci. 2025 Nov 12;26(22):10970. doi: 10.3390/ijms262210970 (PMC12652911; doi:10.3390/ijms262210970)
Supplement: Supplementary file 1 [file ijms-26-10970-s001.zip › 20251114_SupFigs_Resubmission.pdf]

Supplementary Material & Methods

Cell viability assay

Cell viability was measured using the WST-8 assay. For C3H10T1/2 cells, cytotoxicity was assessed over 7 days from differentiation induction to completion of differentiation promotion. For RAW264.7 cells, cytotoxicity was assessed after 24 hours of treatment with the components.

Preparation of Various Extracts

Using hot water at 100°C, extracts were prepared following the same procedure as for the water extract, designated as (CHWE). Using a 4% acetic acid solution, extraction was performed following the same procedure as for the water extract, designated as (CAE). The freeze-dried water extract was redissolved in 100°C hot water, heated for 2 hours, and then freeze-dried again, yielding (HT-CWE). The freeze-dried water extract was redissolved in 4% acetic acid solution and stirred at 4°C for 24 hours. After removing acetic acid using an evaporator, freeze-drying was performed, yielding (AA-CWE).

Oil Red O staining

After washing the fully differentiated adipocytes with PBS (-), 10% formalin solution was added and incubated for 24 hours. Following fixation, formalin was removed from the cells, which were then dehydrated with 60% isopropanol before staining the fat droplets using Oil Red O staining solution.

TG Measurement

After washing fully differentiated adipocytes with PBS (-), methanol and chloroform were added to recover intracellular triglycerides. Following methanol removal, samples were redissolved in 2% Triton X-100 solution, and triglyceride concentration was measured according to the TG kit protocol.

cAMP Measurement

Differentiated adipocytes were treated with isoproterenol, and cAMP concentrations were measured using the cAMP Glo-assay kit, following the manufacturer's protocol.

Measurement of CRE Transcription Activity

pGL4.29 [luc2P/CRE/Hygro] plasmid (Promega K.K., WI, USA) was transfected into different C3H10T1/2 cells using Lipofectamine 2000 (Thermo Fisher Scientific Inc.,

Waltham, MA , USA) following the manufacturer's protocol. Following transfection, cells were selected with 100 µg/mL hygromycin and used as stable expression strains in subsequent experiments. The transfected cells were cultured in a 96-well plate and treated with the extracts and ISO. The luciferase assay (Promega K.K) was then conducted according to the manufacturer's instructions.

Western blotting

Mature adipocytes were treated with ISO at various time points and harvested using sample buffer. Harvested samples were heated at 95°C for 5 minutes to inactivate proteases and phosphatases. Samples were adjusted to equal protein concentrations and subjected to SDS-PAGE using a 12% polyacrylamide gel. Proteins were transferred to a PVDF membrane using a semi-dry system. For p-CREB, 5% BSA was used as the blocking buffer and 5% skim milk was used for CREB. The primary antibodies used were: p-CREB (Cat No. 9193; Cell Signaling Technology, Danvers, MA, USA) and CREB (Cat No. 4820; Cell Signaling Technology) at 1:2000 dilution.

GC-MS

The obtained samples were analyzed using a GCMS-TQ8040NX triple quadrupole mass spectrometer (Shimadzu, Kyoto, Japana) with a Nexis GC-2030 (Shimadzu) equipped with an InertCap 5MS/NP capillary column (0.25 mm ID × 0.25-µm film thickness × 30 m) (GL Science, Inc, Tokyo, Japan). Pure helium was used as the carrier gas. The column temperature was maintained at 80 °C for 2 min, increased to 325 °C at 10 °C/min, and maintained at 325 °C for 6 min. Mass spectrometry was performed by electron ionization (EI), with an ionization voltage of 70 eV, a vaporization chamber temperature of 230 °C, transfer line temperature of 250 °C, and scan rate of 50 m/z to 500 m/z. The GC/MS data were analyzed using the NIST 17 library and a GC/MS solution (Shimadzu).

Supplemental Figures

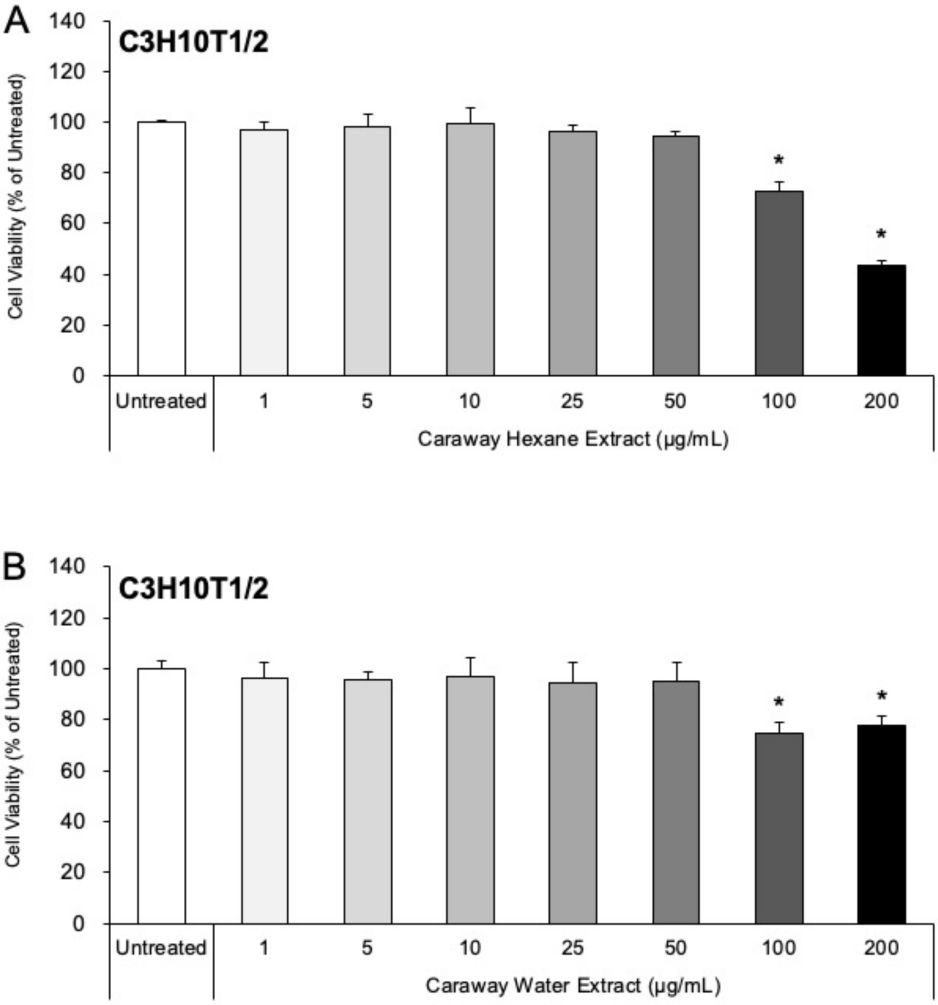

Supplemental Figure S1. Cell viability of C3H10T1/2 adipocytes treated with caraway hexane extract (CHE) or caraway water extract (CWE). C3H10T1/2 adipocytes were treated with CHE (A) or CWE (B) at indicated concentrations for 7 days. The viability of the untreated control was set at 100%, and the relative values are shown as percentage relative to that of the control. Each bar represents the mean ± S.D. (n = 3); (*, $P < 0.05$).

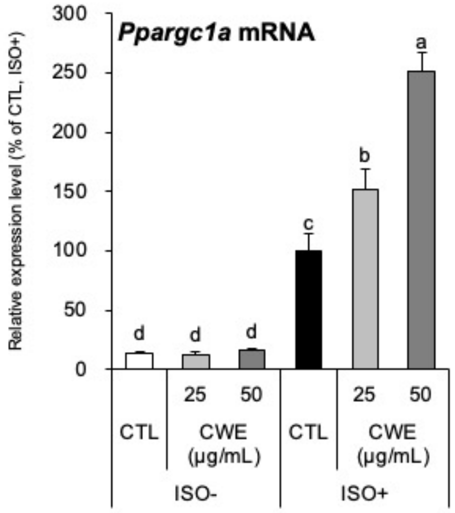

Supplemental Figure S2. Analysis of the effects of CWE on *Ppargc1a*. Analysis of mRNA expression of *Ppargc1a* in C3H10T1/2 cells treated with CHE. The control group was treated with ISO but not with the extract, and mRNA expression in this group was set as 100%, and the relative values of other groups are shown. Each bar represents the mean ± S.D. (n = 3). Different letters indicate statistically significant differences between groups ($P < 0.05$).

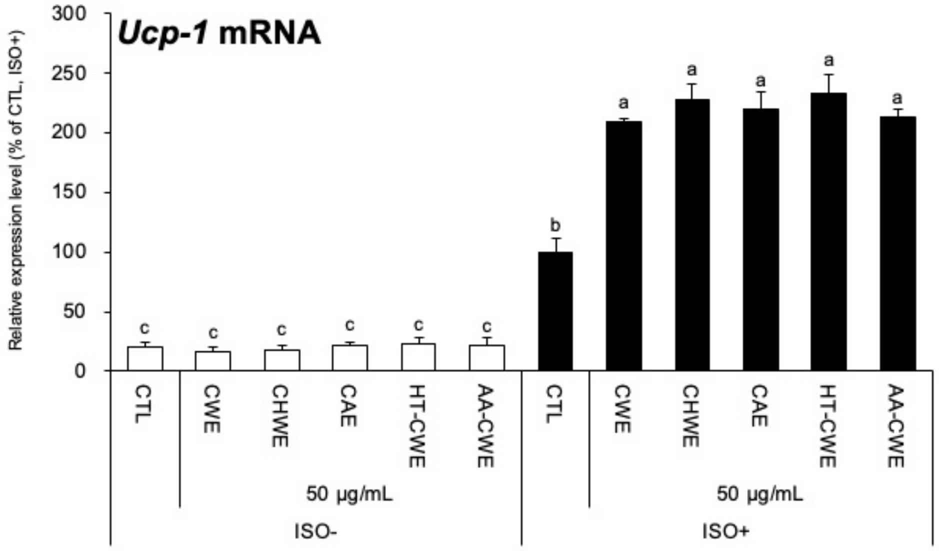

Supplemental Figure S3. Analysis of the effects of samples with different extraction methods and post-processing on *Ucp-1* mRNA expression. Analysis of *Ucp-1* mRNA expression upon ISO stimulation after treating C3H10T1/2 cells with caraway water extract (CWE), hot water extract (CHWE), acetic acid extract (CAE), heat-treated CWE (HT-CWE), or acetic acid-treated CWE (AA-CWE) during induction phase. The control group was treated with ISO but not with the extract, and mRNA expression in this group was set as 100%, and the relative values of other groups are shown. Each bar represents the mean ± S.D. (n = 3). Different letters indicate statistically significant differences between groups ($P < 0.05$).

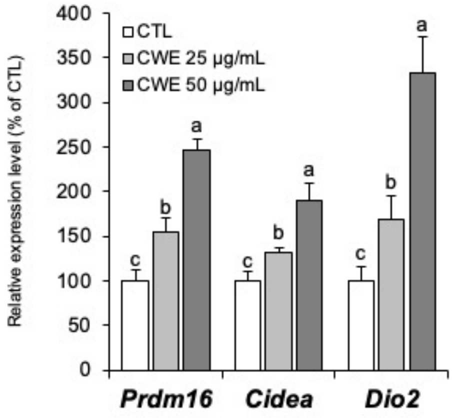

Supplemental Figure S4. Gene expression analysis of beige adipocyte marker genes in CWE-treated adipocytes (with ISO stimulation). mRNA expression in C3H10T1/2 cells treated with CWE. The extract-untreated group was used as the control, set at 100%, and the relative values of the extract-treated groups are shown. Each bar represents the mean ± S.D. (n = 3). Different letters indicate statistically significant differences between groups ($P < 0.05$).

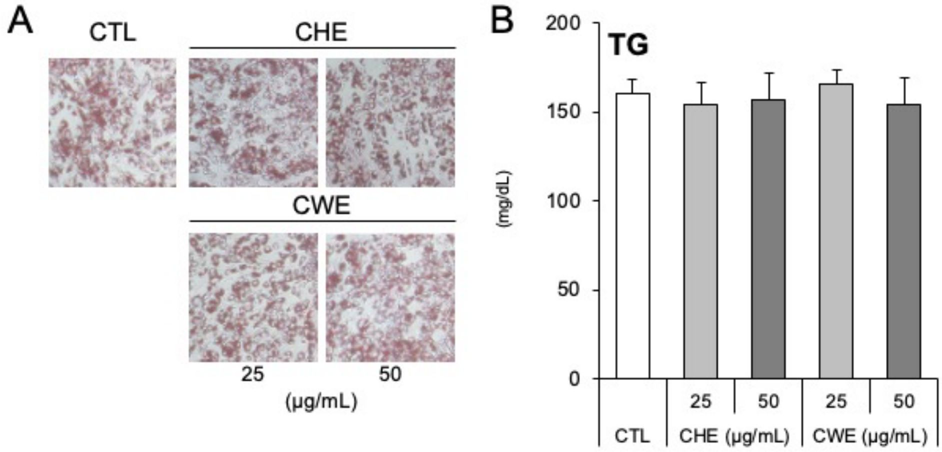

Supplemental Figure S5. Analysis of lipid accumulation in adipocytes treated with CHE or CWE. (A) Oil Red O staining results. Red areas indicate lipid droplets. (B) Triacylglycerol (TG) was extracted from adipocytes, and TG accumulation was measured. Each bar represents the mean ± S.D. (n = 3).

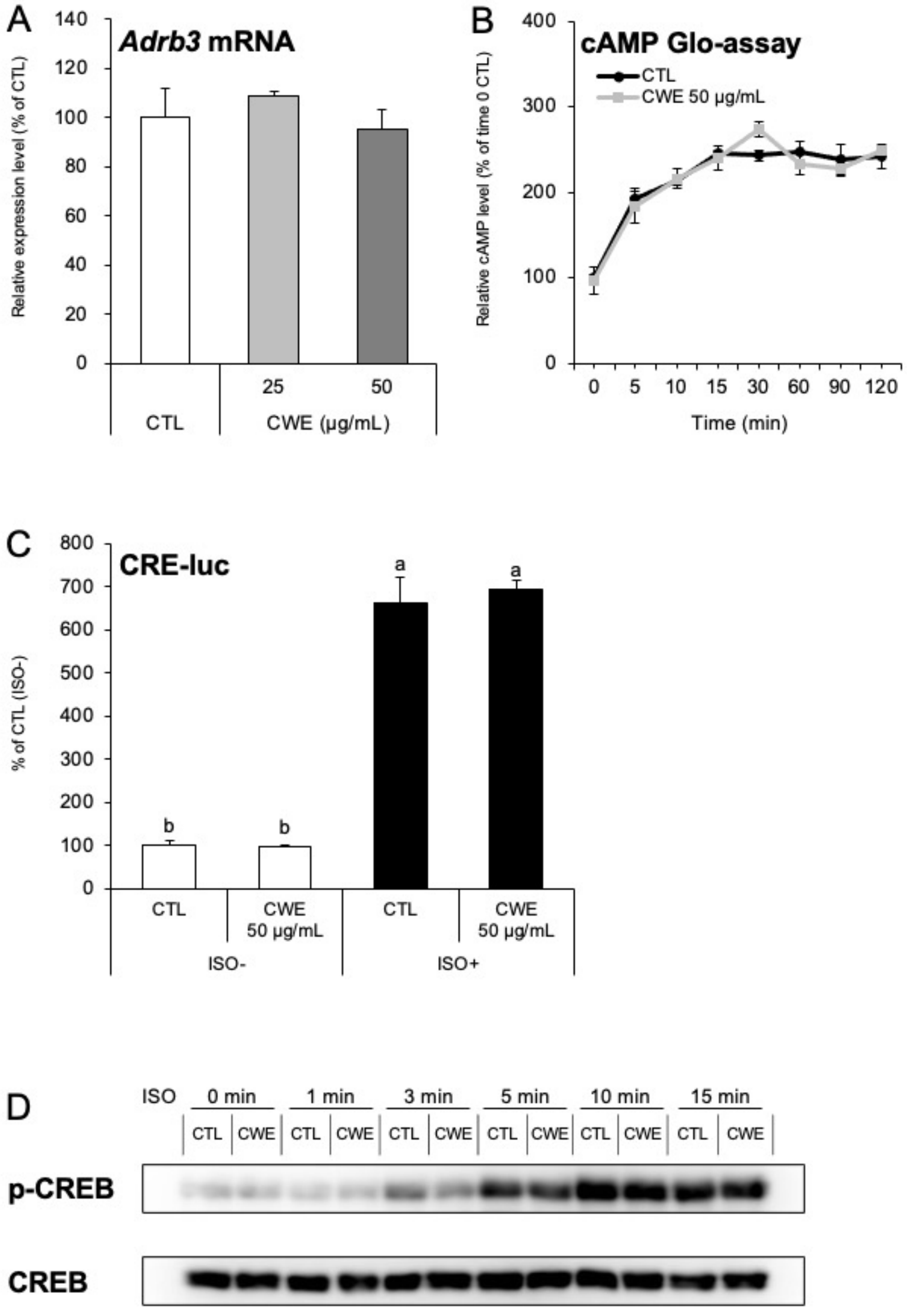
A
Adrb3 mRNA
Relative expression level (% of CTL)
140
120
100
80
60
40
20
0
CTL
25
50
CWE (μg/mL)
B
cAMP Glo-assay
CTL
CWE 50 μg/mL
Relative cAMP level (% of time 0 CTL)
400
300
200
100
0
0 5 10 15 30 60 90 120
Time (min)
C
CRE-luc
% of CTL (ISO-)
800
700
600
500
400
300
200
100
0
a
a
b
b
CTL
CWE 50 μg/mL
CTL
CWE 50 μg/mL
ISO-
ISO+
D
ISO
0 min
1 min
3 min
5 min
10 min
15 min
CTL CWE CTL CWE CTL CWE CTL CWE CTL CWE CTL CWE
p-CREB
CREB

Supplemental Figure S6. The mechanism of CWE-induced enhancement of adrenergic sensitivity in C3H10T1/2 adipocytes treated with CWE during differentiation induction. (A) Analysis of β3-adrenergic receptor mRNA expression. mRNA expression levels were measured under conditions without ISO stimulation. The extract-untreated group was used as the control, set at 100%, and the relative values of the extract-treated groups are shown. (B) cAMP levels were measured after ISO stimulation. (C) Measurement of CREB transcriptional activity using C3H10T1/2-CRE-luc cells. The control group was treated with ISO but not with the extract, and mRNA expression in this group was set as 100%, and the relative values of other groups are shown. (D) Evaluation of CREB activity by western blotting. Phosphorylation was confirmed at 1, 3, 5, 10, and 15 minutes after ISO stimulation. Each bar represents the mean ± S.D. (n = 3). Different letters indicate statistically significant differences between groups ($P < 0.05$).

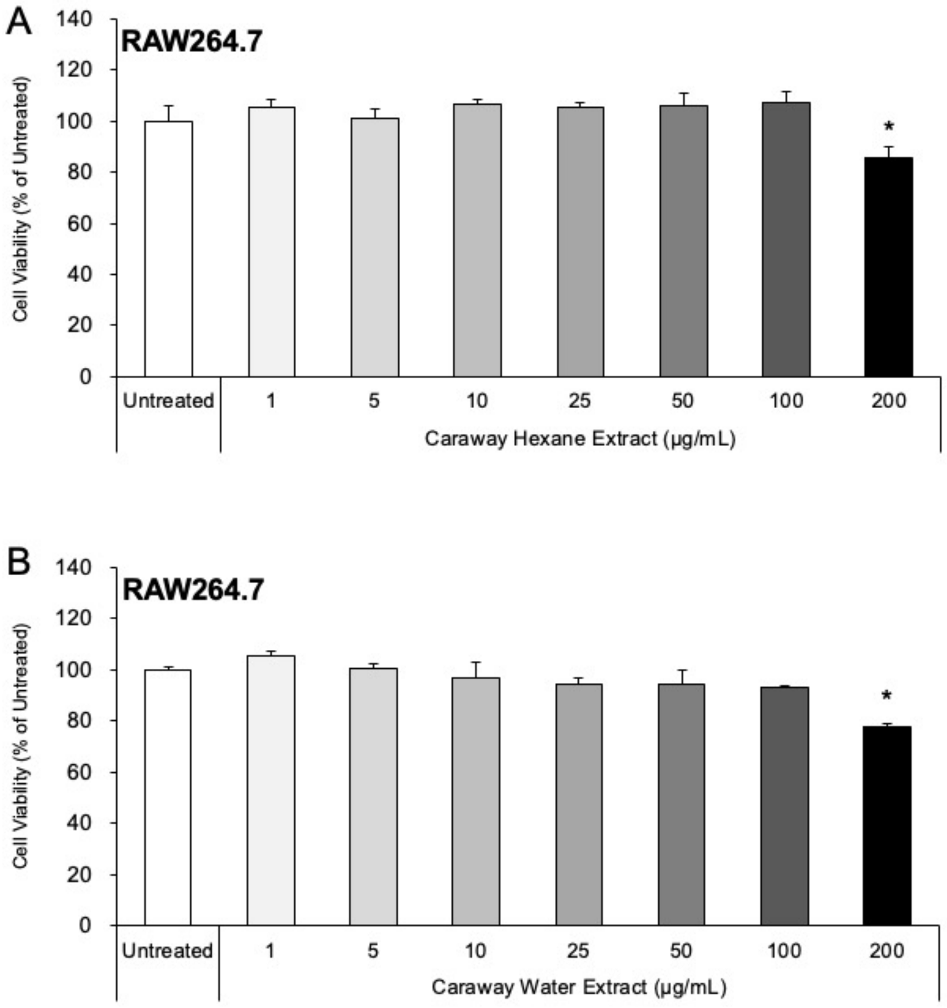

Supplemental Figure S7. Cell viability of RAW264.7 macrophages treated with CHE or CWE. RAW264.7 macrophages were treated with CHE (A) or CWE (B) at indicated concentrations for 24 h. The viability of the untreated control was set at 100%, and the relative values are shown as percentage relative to that of the control. Each bar represents the mean ± S.D. (n = 3); (*, $P < 0.05$).

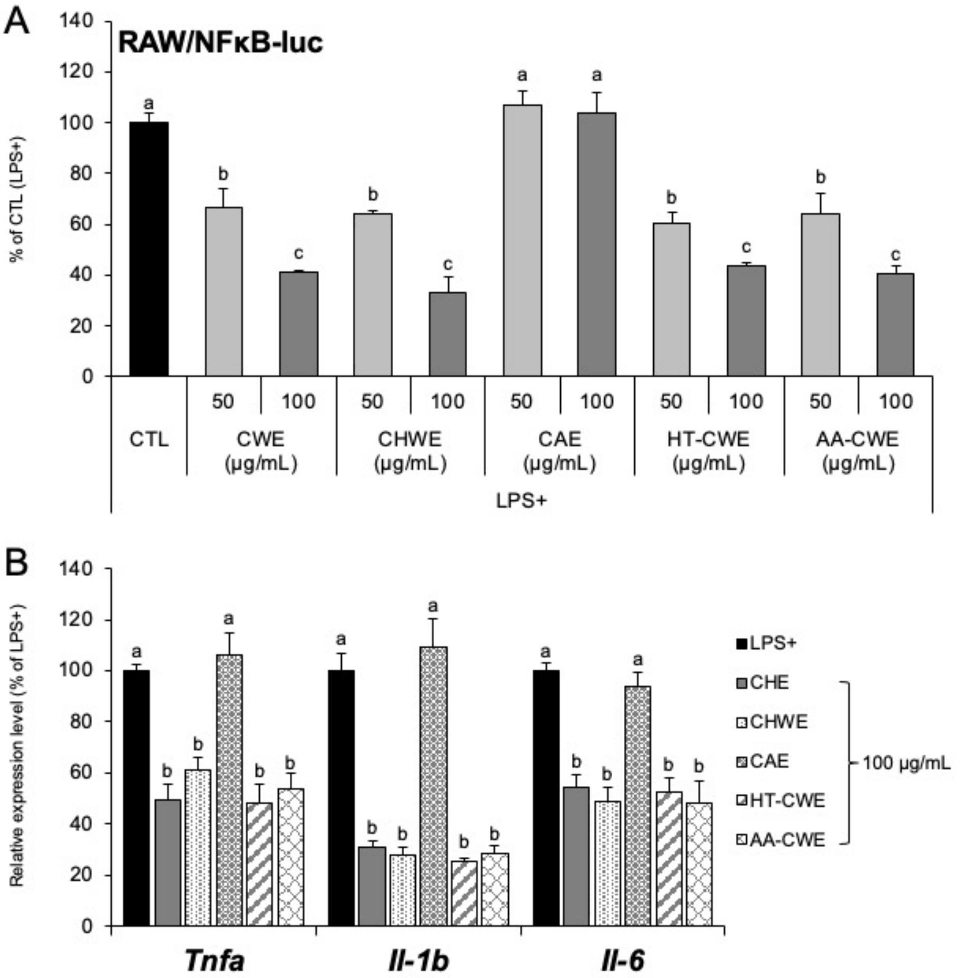

Supplemental Figure S8. Analysis of anti-inflammatory effects in RAW 264.7 cells treated with samples processed using different extraction methods and post-treatments. (A) Anti-inflammatory effects of samples were evaluated using RAW/NFκB-luc with light emission intensity as an indicator. LPS-stimulated and untreated groups were used as controls, and expression was set as 100% and relative values were expressed as a relative percentage. (B) mRNA expression in RAW264.7 cells treated with carvone or limonene. The LPS-stimulated group without any treatment with compounds was used as a control, and expression in this group was set as 100% and the relative values were expressed as a relative percentage of the control. Each bar represents the mean ± S.D. (n = 3). Different letters indicate statistically significant differences between groups ($P < 0.05$).

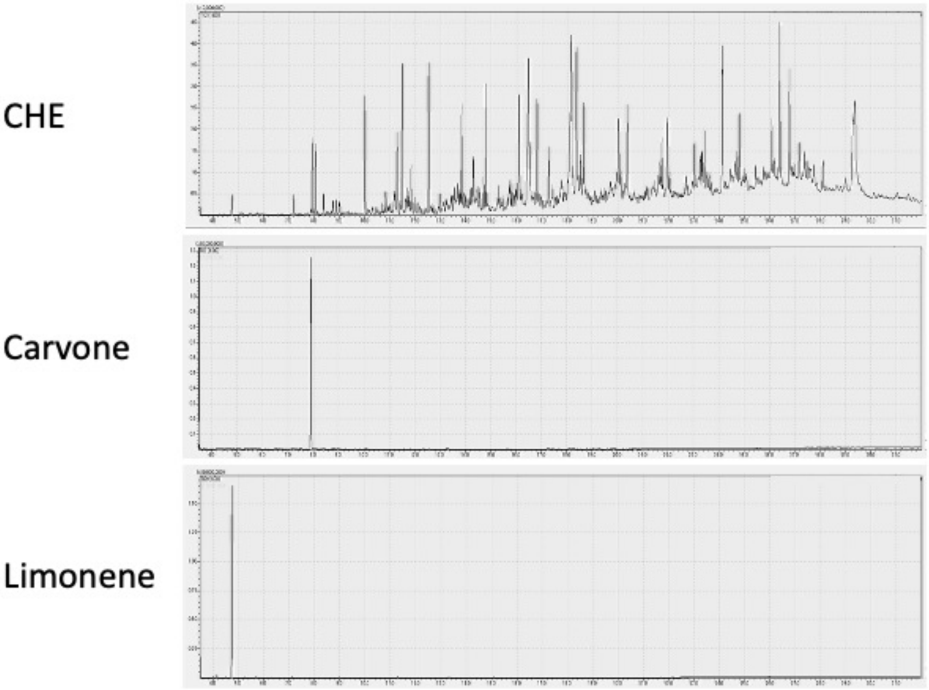

Supplemental Figure S9. GC-MS analysis of CHE. GC-MS analysis was performed to determine the content of carvone and limonene, which have been reported to be present in CHE.

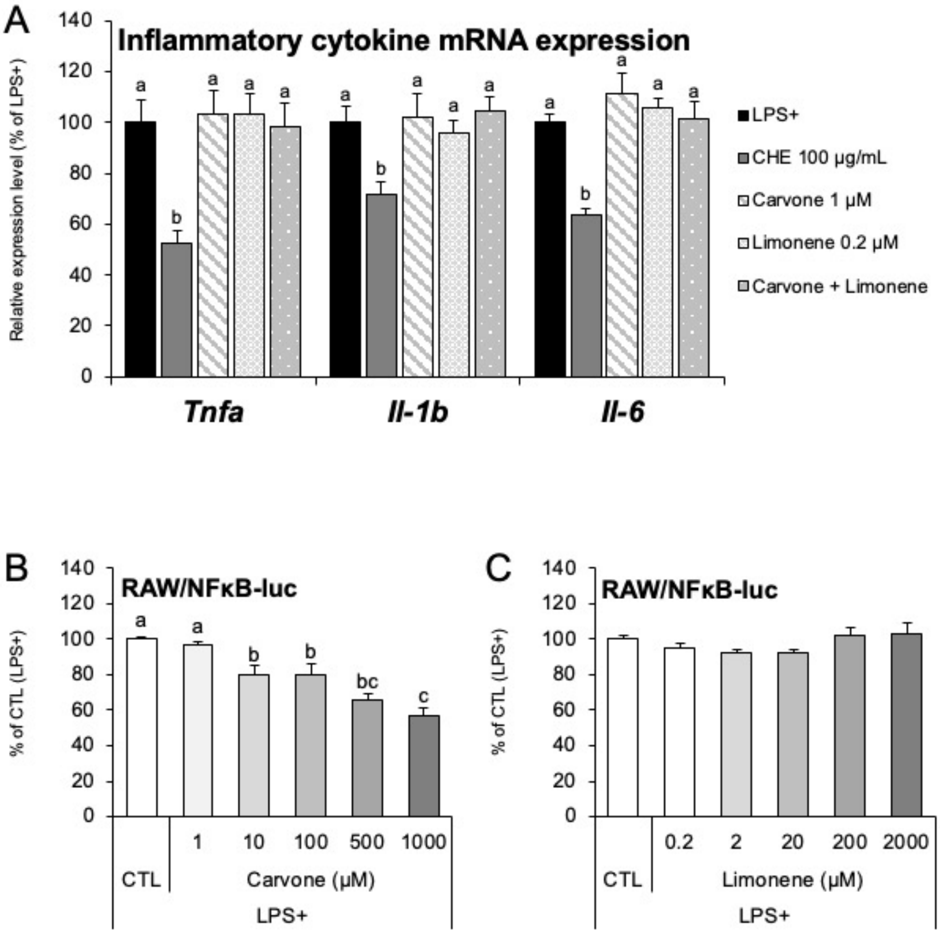

Supplemental Figure S10. Analysis of anti-inflammatory effects in RAW 264.7 cells treated with CHE, carvone, or limonene. (A) mRNA expression in RAW264.7 cells treated with carvone or limonene. The LPS-stimulated group without any treatment with compounds was used as a control, and expression in this group was set as 100% and the relative values were expressed as a relative percentage of the control. (B, C) Anti-inflammatory effects of carvone or limonene were evaluated using RAW/NFκB-luc with light emission intensity as an indicator. LPS-stimulated group and without any treatment with compounds was used as control, and expression in this group was set as 100% and relative values were expressed as a relative percentage. Each bar represents the mean ± S.D. (n = 3). Different letters indicate statistically significant differences between groups ($P < 0.05$).

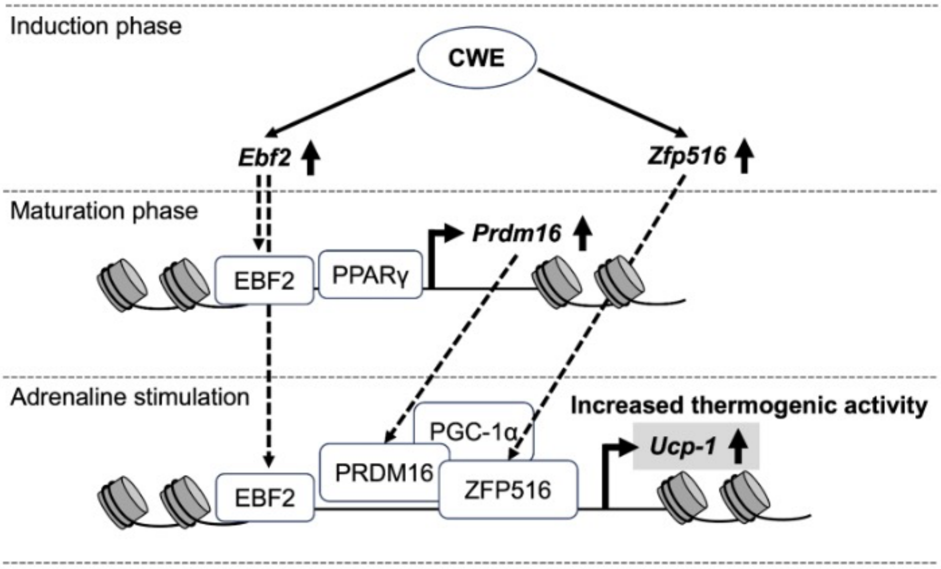

Supplemental Figure S11. Overview of the mechanism by which CWE enhances *Ucp-1* expression. Solid arrows indicate factors affected by CWE. Dashed arrows indicate the mechanism assumed based on these results.

Supplemental Tables

Supplemental Table S1. Primer sets used in the experiment.

Gene name	Forward	Reverse
36b4	CCACCTGGAGAACAACCCAG	CCACCTGGAGAACAACCCAG
Ucp-1	CAGGGAGAGAAACACCTGCC	CATTGTAGGTCCCCGTGTAG
Pparg	GACCCAGCTCTACAACAGGC	CCCAAACCTGATGGCATTGTG
Fabp4	GCAGACGACAGGAAGGTGAAG	GCCTTTCATAACACATTCCACCAC
Adipoq	CTTGTGCAGGTTGGATGGCA	TTAGGACCAAGAAGACCTGCAT
Prdm16	GTCTACGGTGAACGGAAGCC	CCGCTTTTCTACCCTGCTGT
Cidea	AGGCCGTGTTAAGGAATCTGC	GCTGCTCTTCTGTATCGCCC
Dio2	CTCCTAGATGCCTACAAACAGGTTA	GTCAAGAAGGTGGCATTCGG
Cox4	CCGTCTTGGTCTTCCGGTTG	ACACTCCCATGTGCTCGAAG
Cycs	AGGCAAGCATAAGACTGGACC	TCTCCCCAGGTGATGCCTTTG
Ppargc1a	CAGAGTCACCAAATGACCCCA	CAAGAGGGCTTCAGCTTTGG
Tnfa	AGGCACTCCCCCAAAAGATG	CACTTGGTGGTTTGCTACGAC
Il-1b	TGCCACCTTTTGACAGTGATG	GCTCTTGTTGATGTGCTGCTG
Il-6	ACAAAGCCAGAGTCCTTCAGAG	GTGACTCCAGCTTATCTCTTGG
